# Introduction to Psychiatry

**Published:** 2009

**Authors:** R. Srinivasa Murthy

**Affiliations:** Professor of Psychiatry (retd), Bangalore, India. E-mail: Murthy_srinivasar@yahoo.co.in

**Figure:**
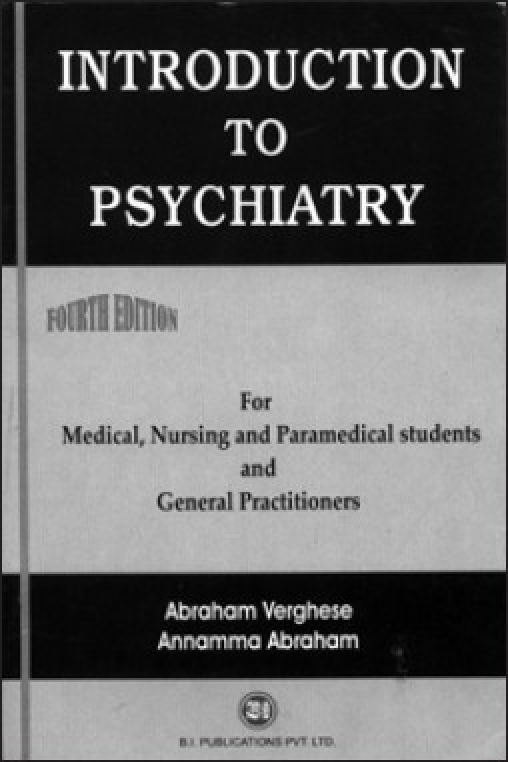


I have known the two authors for over 40 years. They were my first teachers of psychology and psychiatry. I have been reading their Introduction to Psychiatry from the first edition (1976) onwards. The second edition came in 1983, third edition in 1996, and the 4^th^ edition in 2007. In a way, the progress of the book represents the decennial growth of psychiatry as a discipline as well as the growing importance of psychiatry to non-specialist health personnel. Both authors deserve compliments for their commitment to educating the non-specialists and in keeping up with the advances in psychology and psychiatry.

The book has 35 chapters. The first six chapters cover the foundation of psychiatry, in terms of historical aspects, personality, psychopathology, brain and mind, etiology. The next four chapters present the classification of mental disorders, common symptoms, psychiatric examination, and psychological assessment. The following 14 chapters cover the different psychiatric syndromes. The different treatment methods, namely, psychotherapy, physical treatments, and psychopharmacology. The special areas of psychiatry like liaison psychiatry, community psychiatry, psychiatric epidemiology, transcultural psychiatry, alternative medicine, legal aspects are covered in the last section. The book ends with a glossary of common terms, references and a valuable set of multiple-choice questions for the trainees. The index makes the use of the book easy, while looking up a specific topic.

Each of the mental disorders from Chapters 11-24 is considered in detail under the brought headings of definition, etiology and psychopathology, clinical manifestations, differential diagnosis, management followed by key references for further reading. The range of interventions for management is very extensive (either in terms of the range of drugs, or psychosocial interventions). In this process some of the India specific emphasis is missing, for example the importance of family in the treatment is mentioned but not adequately elaborated.

India launched the National Mental Health Programme in 1982 and provided stimulus for a wide variety of non-institutional initiatives. One of the common themes of all these initiatives, whether it is the integration of mental health care with general health care or the different places of mental health care in the community, is the involvement of a wide range of non-specialist personnel for mental health care. In different parts of India, volunteers (suicide prevention, disaster mental health care, drug dependence care), school and college teachers, ex-patients (drug dependence), family members of the persons with mental disorders (schizophrenia, mental retardation, dementia), short-term trained personnel (half-way homes), and health workers and primary care doctors are discharging a wide variety of mental health care tasks. This initiative has met a large gap in the availability of specialist mental health professionals. In this context the book under review will be a resource for these personnel and the trainers of these different categories of personnel.

The book in its current form has one limitation. It is too comprehensive to meet the limited mental health training needs of either the medical students, nursing personnel or paramedical students or general practitioners. The experience of training these personnel teaches us that these groups need ‘focused’ information relevant to the roles they have to play. Further, too much of information, in terms of either theory, or the variety of interventions tends to limit the ease of acceptance of the mental health care responsibilities.

In trying to be comprehensive, this very good book reaches the textbook proportion. In future revisions, it would be more useful to present the information separately for the different groups so that the information can be tailored to the educational background and tasks of the different categories of personnel. The book could also become more user friendly with the use of boxes and summarizing tables to increase interest in reading the book. There are a number of illustrations and more of these would add to the value of the book. The book can be Indianized by referring more details of the Indian research and experiences.

It is very creditable that the two authors have continuously updated the mental health information for non-specialist personnel. This type of an effort in the Indian context is rare. They deserve appreciation for the same.

I have enjoyed reading the book and recommend strongly for inclusion in all the libraries of medical colleges, nursing colleges, and training centers of health personnel of all categories.

